# "I am nurse, I am partner, I am cook – I am everything..." roles and functions of relatives in supporting therapy adherence and abstinence in patients with alcohol-related liver cirrhosis prior to listing for liver transplantation: a qualitative analysis

**DOI:** 10.1186/s13722-026-00673-3

**Published:** 2026-05-11

**Authors:** Annette Binder, Julia Fenchel, Immanuel Lang, Anil Batra

**Affiliations:** 1 Addiction Medicine and Addiction Research Section, Department of General Psychiatry and Psychotherapy, University Hospital Tübingen, Calwerstraße 14, 72076 Tübingen, Germany; 2 German Center for Mental Health (DZPG), Partner Site Tübingen, Calwerstraße 14, 72076 Tübingen, Germany

**Keywords:** Liver transplantation, Alcohol-related liver disease (ALD), Family involvement, Caregiver role, Therapy adherence, Abstinence maintenance, Social support, Health literacy, Transplant listing process, Psychosocial factors

## Abstract

**Background:**

Alcohol-related cirrhosis is an important indication for liver transplantation (LTX) in Germany and worldwide. In Europe, the USA, and Canada patients with alcohol-related cirrhosis often have to maintain at least six months of abstinence and demonstrate treatment adherence before being listed for transplantation. Nevertheless, country-specific exception policies may allow earlier liver transplantation in selected cases. These requirements, combined with the severity of the disease, create challenges for patients, their relatives and the healthcare system. This study aimed to examine the roles and functions of family members in supporting adherence to therapy and maintaining abstinence in patients with alcohol-related cirrhosis prior to LTX listing.

**Methods:**

A total of 35 interviews were conducted, including narrative interviews with 10 relatives, 11 patients, and 3 former patients, as well as semi-structured interviews with 11 healthcare professionals from different LTX centers across Germany. Interviews were audio-recorded, transcribed, and analyzed using qualitative content analysis.

**Results:**

Relatives played a central role in supporting therapy adherence and abstinence, particularly by actively assisting with the implementation of treatment recommendations. They also assumed significant responsibility for acquiring and applying health literacy. Additionally, family members provided critical emotional support, yet their own psychosocial needs were often overlooked, leaving them without sufficient resources to cope with the associated burdens.

**Conclusions:**

Building on our findings, we propose a family-centered integrated model for patient-centered care in advanced liver disease and LTX. This model emphasizes the recognition and support of family members in clinical practice, aiming to improve both patient outcomes and the well-being of relatives involved in the care process.

**Supplementary Information:**

The online version contains supplementary material available at 10.1186/s13722-026-00673-3.

## Introduction

End-stage chronic liver damage due to alcohol-related cirrhosis is a life-threatening condition for which liver transplantation is often the only viable treatment [1]. Unfortunately, organ scarcity means that not all patients who have an indication for a liver transplant actually receive an allograft. Liver transplantation (LTX) for alcohol-related cirrhosis has a favorable outcome, comparable to that of other causes of end-stage liver disease [2, 3]. This patient group faces specific regulations regarding access to transplantation. Currently, in Germany and many other countries, patients with alcohol-related liver disease (ALD) are required to demonstrate abstinence for 6 months before being listed for LTX [4–7]. Nevertheless, several countries apply specific exception criteria that enable earlier liver transplantation based on individual clinical and psychosocial risk assessment.

While abstinence from alcohol can promote partial regeneration of the liver, it remains crucial even after transplantation to avoid harm to the graft. In patients with alcohol-related cirrhosis, the underlying condition involves alcohol dependence syndrome (ICD-10 F10.2) or harmful alcohol consumption (ICD-10 F10.1) [8]. Successfully achieving and maintaining abstinence requires psychoeducational counseling, motivational support using targeted techniques, and, if necessary, a medication-assisted withdrawal treatment [9], which can be challenging in patients with cirrhosis [10]. Therapeutic strategies, including cognitive behavioral therapy and, when necessary, anticraving pharmacotherapy, can facilitate long-term stabilization [11, 12]. Ultimately, integrated care models that foster close collaboration between addiction specialists and hepatologists or other medical professionals involved in the treatment of liver disease are essential for improving outcomes in this patient population [13]. Additional benefits can be achieved through psychosocial support provided by the patient’s social environment, including family members [14], while social isolation has been identified as a risk factor for post-transplant relapse [15].

As part of the psychosocial assessment, healthcare professionals have the task of assessing the support provided by the patient’s family members and caregivers. National and international treatment guidelines for liver transplantation recommend that psychosocial evaluation, including assessment of social support, should be prioritized at the beginning of the evaluation process in patients with harmful alcohol consumption, addiction disorders, or concerns regarding adherence, with further evaluation potentially postponed when substantial concerns regarding abstinence or adherence persist [16, 17]. Some practice guidelines additionally highlight adequate social or caregiver support as an important component of patient care, both during waitlisting and during postoperative recovery [6]. This underscores the central role of family and caregiver support in the evaluation and long-term success of liver transplantation. Inadequate abstinence or non-adherence may also lead to non-approval for listing, which may result in delays or even discontinuation of the transplant evaluation process, thereby reducing the likelihood of receiving a liver transplant. The recommendations primarily emphasize the assessment of social support, while the role of HCPs in actively strengthening social support, identifying potential unmet needs among relatives that could jeopardize long-term support, or enabling relatives to take on a supportive role is not a central focus.

The involvement of relatives plays a crucial role in the care of patients with end-stage diseases, including those awaiting liver transplantation. As both emotional anchors and practical caregivers, family members can significantly influence clinical outcomes and the patient’s ability to cope with the challenges of illness and treatment. There is evidence that psychosocial and family support has a positive impact on post-transplant outcomes [18] including abstinence [15]. At the same time, relatives often face a multitude of responsibilities and emotional burdens, which can place considerable strain on their own well-being. These stressors may, in turn, impair the patient’s coping capacity, mental health, and overall quality of life [19]. These findings highlight the importance of systematically including relatives in comprehensive treatment concepts for end-stage liver disease and liver transplantation, recognizing their pivotal role and addressing their psychosocial needs as an integral part of patient care.

This study employs the Integrated Model of Advanced Liver Disease (IMALD) by Naik et al. [20] as a sensitizing concept. Sensitizing concepts provide a general sense of reference that guides attention towards potentially relevant phenomena [21]. The IMALD provides a comprehensive lens for understanding and addressing the complex interplay of factors impacting patients with advanced liver disease. This model emphasizes the integration of medical, psychological, and social dimensions to optimize care and improve patient outcomes. It highlights that advanced liver disease is not merely a medical condition but one deeply influenced by psychosocial stressors, health behaviors, and systemic challenges. A crucial component of the IMALD is the role of family members and caregivers in supporting the patient. By incorporating family dynamics and caregiving roles, the IMALD acknowledges the critical contribution of relatives to the overall success of treatment strategies [20].

Developed in recognition of significant gaps in supporting psychosocial approaches for relatives, our study focuses on how three elements, including the role of relatives in maintaining patients' therapy adherence and abstinence and unmet psychosocial needs of relatives to be able to support patients as well as HCPs’ perspectives on relatives in the process of preparing for liver transplantation. To fill this research gap, our study was conceptualized to shed light on the roles and functions of family members in therapy adherence and abstinence to potential liver transplantation recipients. With this, we will answer the following research questions:

- What roles and functions do family members take on in the process of preparing for an LTX?
- Which aspects of these functions and roles are relevant for maintaining adherence and abstinence from alcohol?
- What are unmet needs of family members that are important to maintain supporting roles and functions for those affected?

## Materials and methods

### Research design

We applied a qualitative research approach to explore the role of relatives in therapy adherence and abstinence maintenance in patients with alcohol-related cirrhosis before LTX listing, analyzing communication patterns on roles and functions of relatives and patients via narrative interviews. To triangulate and enrich data while ensuring rigor [22], we also conducted semi-structured expert interviews with HCPs from transplant centers to assess family function. For transparency, additional methodological details are available in Supplement 1 via the COREQ checklist [23].

### Ethical considerations

The study was approved by the Ethics Committee of the University of Tübingen (project 718/2020BO2). Participants gave written informed consent. Confidentiality was ensured.

### Recruitment, participant selection and setting

The study was conducted at the Section for Addiction Medicine and Addiction Research at the University Hospital in Tübingen (Germany). Participants—patients with alcohol-related cirrhosis and an indication for liver transplantation, as well as family members—were recruited at the liver transplantation outpatient clinic (University Hospital Tübingen, Psychiatric Clinic). Former patients were informed about the study during routine telephone calls intended to maintain outpatient contact and offer further treatment if needed. HCPs were recruited via e-mail and telephone from all 22 German transplant centers performing LTX at the time of data collection. We included 11 HCPs from 10 different LTX centers.

### Data collection

Narrative interviews were conducted with patients and their relatives. An interview guide structured the semi-structured HCP interviews. All interviews took place from November 2020 to April 2021. To ensure independent perspectives, patients and their family members were interviewed separately in one-on-one settings. Interviews lasted between 18 and 75 min. All interviews were audio-recorded, transcribed verbatim, and anonymized. Recruitment was stopped once thematic saturation was reached [24], which means at the point interviews no longer yielded substantially new findings to our research question. Only recruitment for former patients stopped before this point because there were no more potential participants who agreed to participate.

### Analysis

We analyzed transcripts using qualitative content analysis [25] —often referred to as thematic analysis outside Europe [26]—with data-driven coding, supported by MAXQDA software. Two codebooks were created due to the different perspectives of HCPs and those affected (or their relatives). A cross-analysis was carried out with the focus on the importance of relatives in therapy adherence and maintenance of abstinence, developing thematic concepts and then comparing these thematic concepts across selected codes. Within this, the following codes were used: From the codebook for patients and relatives we used the main category “relatives”, the category “alcohol abstinence” as well as the category “private support”. From the codebook for HCPs, we use the categories “relatives” and “external anamnesis of relatives”. By selecting these codes for cross analysis, 659 of the 1116 coded sections were included in the cross analysis. The themes developed in the cross analysis, along with their definitions and linkage to the categories from the codebooks, are available in Supplementary Table 2 (Themes, Definitions, and Codebook Categories). Details of the analytic process, including data-driven analytic steps and reflexivity considerations, are provided in Supplementary Table 1 (COREQ checklist).

### Trustworthiness of the study

To ensure the trustworthiness of this qualitative study, we adhered to the framework for qualitative rigor outlined by Lincoln and Guba [27], encompassing credibility, dependability, confirmability, transferability, and authenticity. Credibility was enhanced through methodological and data triangulation. We included multiple perspectives by interviewing patients, former patients, relatives, and healthcare professionals from different liver transplant centers. Dependability was addressed by applying a transparent and systematic analytic process based on qualitative content analysis. Confirmability was strengthened through investigator triangulation and reflexive research practices. Coding and interpretation were conducted by two researchers and discussed within the interdisciplinary research team. Transferability was supported by providing a detailed description of the study context, including the German liver transplantation system, legal and organizational frameworks, participant characteristics, and settings. Authenticity was addressed by presenting a balanced and nuanced account of the different perspectives involved. The analysis highlights not only the supportive roles of relatives but also their burdens and unmet needs, as well as ambivalent views expressed by patients and healthcare professionals. By representing converging and diverging experiences, the study aims to provide a fair and authentic portrayal of the complexities surrounding therapy adherence and abstinence prior to liver transplantation.

### Participant characteristics and demographics

The patient sample included 11 individuals in preparation for listing for liver transplantation (LTX) who were undergoing formal proof of abstinence, and 3 individuals who decided to discontinue this process. The relatives sample included 10 independent individuals. Additionally, 11 HCPs who were responsible at the transplant centers for the pre-transplant evaluation and/or for conducting abstinence verification prior to listing for LTX were included. For participant characteristics see Table 1.

**Table 1:** Participant characteristics and demographics

Characteristic	Value
Patient characteristics (*n* = 14)	
Age, mean (range), years	54.1 (34–72)
Female, n	6
Male, n	8
Status in transplant evaluation*	- In preparation/listed: 11 - Discontinued profe of abstinence: 3
Relatives (*n* = 10)	
Age, mean (range), years	55.4 (35–73)
Female, n	5
Male, n	5
Relationship to patient	- Life partner or spouse: 7 - Parent: 1 - Child: 2
HCPs (*n* = 11)	
Age, mean (range), years	46.0 (34–64)
Overall professinal experience (mean, range)	11.0 (6–30)
Professional experience in alcohol-related cirrhosis care, years (mean, range)	6.5 (2–17)
Senior clinical leadership roles	7 (64%)

Note. *For all patients, the indication for LTX was established and the listing evaluation process had been initiated or completed

## Results

**Fig. 1:**
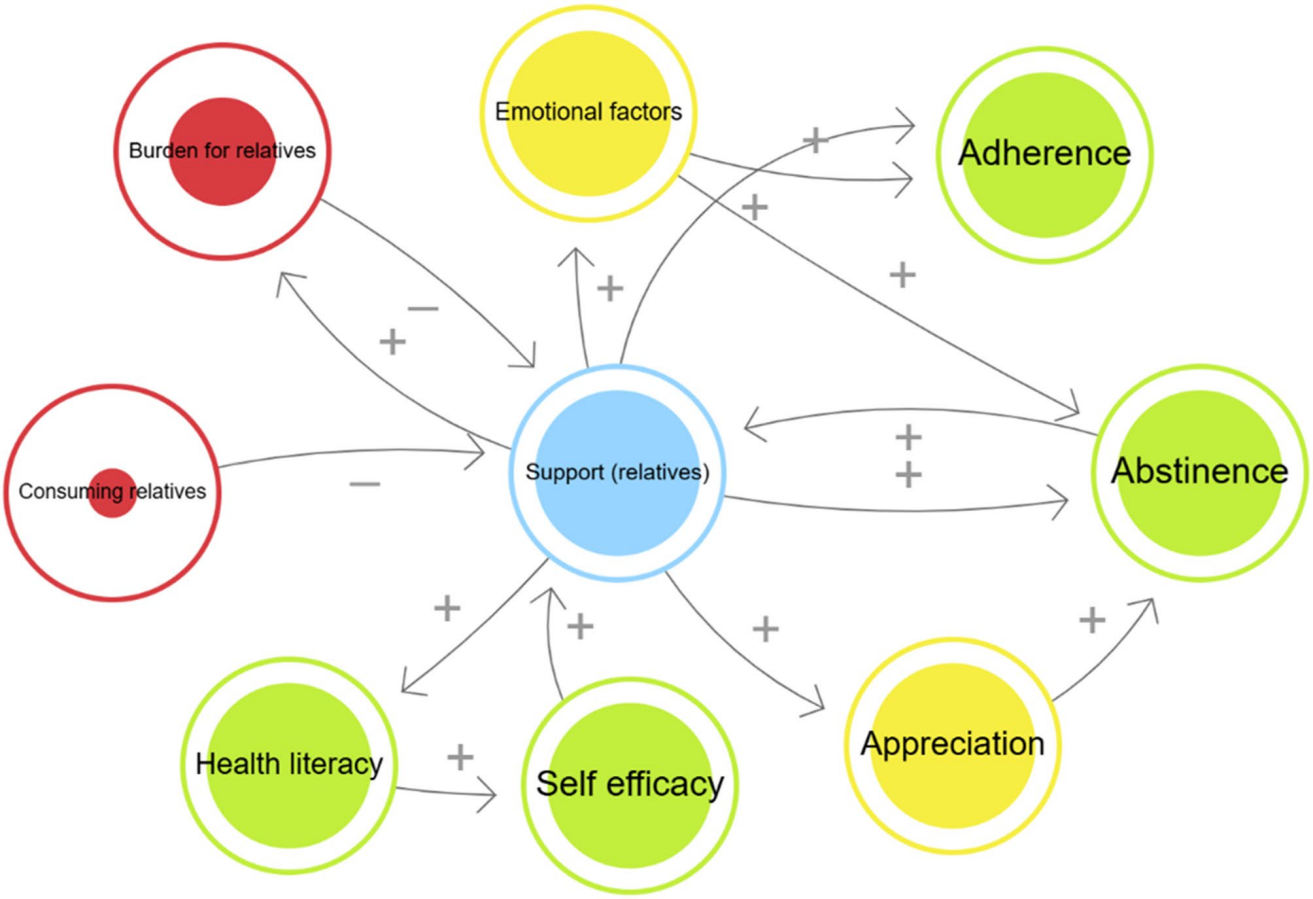
Presentation of selected factors influencing the support of relatives as well as adherence and abstinence

The following section presents the results of the in-depth cross-analysis with a focus on the roles of relatives. Illustrative quotes were selected to provide insight into the material. Selected quotes are presented in the results section to illustrate key themes. Further supporting quotes are available in Supplementary Table 3 (Additional Quotes). Figure 1 provides an overview of the factors identified in the analysis as influencing the supportive role of relatives and the adherence and abstinence of those affected.

### Implementation and maintenance of therapy adherence

The analysis revealed that relatives assume a wide range of responsibilities in the domain of treatment adherence. Their focus frequently includes administrative tasks, such as arranging and supporting the keeping of appointments. Moreover, they play a crucial role in implementing therapy recommendations in daily life, which encompasses managing medication, adhering to dietary requirements, and supporting the maintenance of alcohol abstinence.

*Preparing the medication and*,* yeah*,* you have to keep an eye on everything.* (15 wife)

Those affected emphasize their gratitude for the support, stating that, from their perspective, they would not have been able to implement the necessary changes in daily life on their own.

*That’s when I said: Yes*,* I wouldn’t have made it on my own. If I had been completely alone*,* I wouldn’t have made it.* (8 patient, in treatment)

### Acquiring and supporting health literacy

Regarding health knowledge, it became evident that many relatives had taken on this responsibility themselves. They familiarized themselves with numerous medical topics related to cirrhosis and LTX, and also endeavored to understand the organizational structures of the healthcare system, particularly focusing on LTX. Some relatives also reported that the active acquisition of knowledge helped them feel self-efficacious. The belief that they could contribute to improving the health of the affected individual through their own efforts in acquiring health knowledge appears to play a significant role in this process.

*Then I immersed myself in the topic*,* started making calls everywhere*,* and also contacted the doctors I know to ask for advice on the next steps… and so on. […] For me*,* it was really good to take action and engage with the topic — it gave me a bit of a feeling that I could actually do something.* (16 child)

Those affected appeared to take a more passive role in acquiring health knowledge. Many expressed that they were happy to leave the active acquisition of health information or communication with HCPs to their relatives.

*And of course*,* I’m really glad that my wife took charge of everything. She’s probably going to become a liver specialist one day.* (15 patient, in treatment)

### Family as a motivating element and future perspective to promote abstinence and adherence

A key motivating factor for alcohol abstinence and treatment adherence was the desire to be present for one’s family in the future and to continue spending time with loved ones. Children and grandchildren were especially significant, while partners also played a role. In contrast, individuals who received support primarily from their parents rarely mentioned the motivation of wanting to be there for their parents in the future.

*And I have absolutely no desire to drink anymore. Of course*,* I also started thinking about my children and grandchildren. If things had continued the way they were*,* I wouldn’t have been around to see them grow up. That really had a big impact on me.* (15 patient, in treatment)

Relatives expressed their appreciation for the decision and commitment to abstinence. At the same time, they provided support by removing alcohol from the household or refraining from drinking in the presence of the affected individual.

### Emotional support and relationship

In particular, those affected emphasized the great relevance of emotional support from relatives. In particular, the unconditional support experienced on the way to listing and while being listed for LTX and the open communication about emotions were mentioned.

*Yes*,* and everyone supports me. […] My family called me every day to check on me and see how I was doing… They all worry*,* and we are always in touch. We are always talking to each other — family is family*,* after all.* (17 patient, discontinued treatment)

In some cases, an improvement in open communication and the relationship was even described after the diagnosis was made. This assessment was shared by both those affected and their relatives.

*My relationship with my wife is now so much better than it was before. It’s hard to explain*,* but that’s just how it is — despite all the difficulties*,* you know?* (1 husband)

Adult children who care for their affected parents often mention a change of role in relationships. They have the impression that they have to be emotionally strong for the parent and represent their interests, a role that had been assumed by the parent before the diagnosis. After the diagnosis, this role was perceived as shifted to the children. The affected individuals themselves did not address the issue, but the children mentioned that this role change was also perceived by their affected parents.

### Unmet Needs of family members

Relatives stated that they experience the support of the affected person as a burden due to the high time expenditure, which leads to restrictions in their personal life. Many said they would like to escape the situation for a short time, e.g. for a vacation. Parents who are older themselves stated that their own quality of life is limited due to the situation with an affected child.

Relatives also stated that their own energy resources were reaching their limits due to the wide range of tasks and obligations.

*Because I know that my energy is limited*,* too.* (11 wife)

The affected persons themselves stated that they saw their relatives “sacrificing” themselves for the person concerned, but seemed to accept this uncritically or even welcome it.

*She [wife] really sacrifices a lot. But she does it wonderfully.* (15 patient, in treatment)

The relatives stated that they lacked support for organizational aspects, but that they would also have liked emotional support in the form of supportive conversations for relatives.

### Perspective of HCPs on roles and functions of relatives in the process of transplant preparation

HCPs regard the role of relatives and the associated desire for a meaningful life with the family as key motivators for maintaining abstinence. They also highlight the importance of recognizing and valuing the alcohol abstinence achieved by family members as a crucial supporting factor. Some HCPs noted that relatives who regularly consume alcohol themselves and have limited understanding of the challenges of maintaining abstinence may be less capable of providing effective support. HCPs also emphasized the appreciation expressed by relatives for the patient’s achieved and maintained abstinence, as well as the crucial support provided by family members. These aspects were perceived by HCPs as key factors influencing the decision for LTX.

*Of course*,* relatives provide stabilizing support. […] As a family*,* they are very proud of you because you no longer drink. And they are very happy*. (1HCP surgeon)

Furthermore, pre-LTX support from relatives was considered a strong indicator of continued support post-LTX. This is particularly relevant from the HCPs’ perspective, as long-term adherence to medication, regular check-up appointments, and sustained alcohol abstinence remain critical even after LTX.

*It is extremely important because they should – hopefully – support abstinence and also assist the patient in the postoperative phase. If not relatives*,* then at least close friends or another social network. After the operation*,* patients are physically weakened*,* need to take medication consistently*,* and must remain abstinent in the long term.* (8HCP psychosomatic physician)

On the one hand, HCPs perceive the relatives’ desire for the affected individuals to receive timely and high-quality treatment as a positive factor, as it indicates their active involvement in providing support and care. On the other hand, they also recognize the significant burden this means for relatives, particularly children of those affected. According to some HCPs, relatives frequently accompany patients to the LTX center for appointments. However, they report that therapeutic or supportive conversations for relatives are generally not offered at their centers. While some HCPs believe that relatives would likely benefit from additional support, others state that attempts are made within patient care to address their most immediate stressors. Nevertheless, HCPs commonly observe that relatives themselves are often in a state of crisis and may struggle to recognize their own needs without professional guidance. Children of those affected are seen as being particularly burdened.

*When relatives come to us*,* they are often in a state of crisis themselves and are often unable to take care of their own needs.* (6HCP psychologist)

## Discussion

This study identified multiple dimensions of the role of family members in providing supportive functions, both in the practical implementation of therapy adherence and in influencing various factors related to alcohol abstinence. Additionally, potential negative impacts on the supportive role were highlighted, including the strain of the situation and the associated responsibilities, unmet needs of family members, and the family members’ own substance use.

The practical tasks undertaken by family members played a particularly important role in supporting therapy adherence and influencing various factors related to motivation for abstinence. A similar picture emerged in a qualitative study conducted after early liver transplantation in individuals with alcohol-related cirrhosis [28]. Participants reported experiencing difficulties in navigating the medical system to obtain the correct diagnosis or treatment, both prior to liver transplantation and during the six-month abstinence detection phase. While some participants described receiving support from their treatment team in finding and referring them to a transplant center, others reported having to undertake the search themselves, which often resulted in delays in the referral [28]. Our study highlighted that family members play a crucial role in the practical implementation of therapy adherence. This includes not only ensuring that medical appointments are kept but also preparing medications. Managing medical treatment as part of self-management before and after liver transplantation was also identified as an important aspect in a qualitative study, in which family members were found to be essential in supporting this process [29]. Similar to our study, a study focusing on the post-transplant period found that family members played a key role in the practical implementation of dietary recommendations after liver transplantation, such as shopping and cooking [30].

Furthermore, some participants described their families’ encouragement as the primary driver of their dietary intake [30]. In our study, family members often engaged intensively with health-related knowledge about liver transplantation, demonstrating a high level of health literacy. On the one hand, this seemed to empower them to provide the best possible support for the affected individuals. On the other hand, it appeared to serve as a way for them to experience a sense of self-efficacy. In contrast, the patients themselves tended to remain more passive, which could be related to an avoidance of directly confronting the potential consequences of their illness or to cognitive limitations caused by the disease. A study found that patients with ALD had critical misconceptions, omissions, and a general lack of understanding regarding their condition and its treatment [31]. Another study has shown that for organ transplant recipients (without focusing on alcohol-related cirrhosis), health literacy is a key factor in therapy adherence [32]. An investigation into the subscales of the health literacy questionnaire revealed that ‘Social support for health’ was a significant influencing factor [32]. This underscores the important role of family members in the acquisition of health literacy related to liver transplantation. A crucial component of the IMALD is the involvement of family members and caregivers in patient support. They provide emotional and logistical assistance, promote adherence to treatment, and help navigate the complexities of liver disease management [20]. However, in the original model, the role of relatives is positioned as supportive alongside the patient, rather than as a central element. Building on this foundation, our findings led us to expand the IMALD model by emphasizing the central role of relatives as “Informed, Supporting & Well-Supported Relatives.” This addition highlights the importance of equipping family members with adequate information, enabling them to provide effective support.

Our study showed that positive recognition from relatives for the abstinence achieved was experienced as a motivating factor by those affected. This aligns with family members’ perception of positive reinforcement as a key element of the Community Reinforcement and Family Training (CRAFT) approach, which supports behavioral change while maintaining relational stability [33].

This suggests that marriage or close family relationships may have a protective effect on multiple levels. At the same time, successful abstinence can foster a sense of self-efficacy among relatives, as they perceive their support as contributing to this success. In contrast, relapses may be associated with feelings of helplessness and reduced self-efficacy among family members. As a result, potential relapses may be seen as a dual burden on the family system, placing significant stress on relatives.

Managing various emotions can be challenging and, in some cases, may increase the risk of alcohol relapse. In a qualitative study, the entire process leading up to LTX was described as an “emotional rollercoaster” [34]. During the recovery phase in particular, recipients experienced unexpected waves of emotions, which were supported and, in some cases, buffered by their relatives. Family members also reported that their loved ones became more emotionally reactive, both in positive and negative ways. Considering the findings of our study, it can be inferred that relatives who are well integrated into the support of LTX recipients and receive assistance in addressing their own needs may be better equipped to handle the emotional challenges of the critical postoperative phase in a way that fosters both support and abstinence. Social interaction and emotional support are fundamentally important for human well-being. During challenging life events such as serious illness, these forms of support gain particular relevance. A single study has indicated that patients on the liver transplantation waiting list due to alcohol-related cirrhosis—regardless of coexisting viral factors—reported lower levels of perceived social support [35]. In light of our findings, it can be assumed that insufficient or low perceived support may hinder patients’ ability to cope with the psychological burden of being on the transplant waiting list.

Our study highlighted the notable burdens and personal constraints experienced by relatives. At the same time, HCPs emphasized that they frequently observe relatives in a state of crisis, struggling to address their own needs and potentially requiring professional support. Consistent with these findings, a study on the well-being of caregivers of individuals with end-stage liver disease found that many relatives experienced diminished well-being [36]. The credibility of these findings is supported by the multi-perspective qualitative design and the systematic analytic approach applied in this study.

Considering these unmet needs alongside the critical role of family members highlighted in our study, it becomes evident that relatives must be actively integrated into the care of patients in accordance with the bio-psycho-social model. For patients without close relatives, these supportive functions may need to be provided by professional or peer-based support structures, underscoring the need for flexible, inclusive care pathways.

**Fig. 2:**
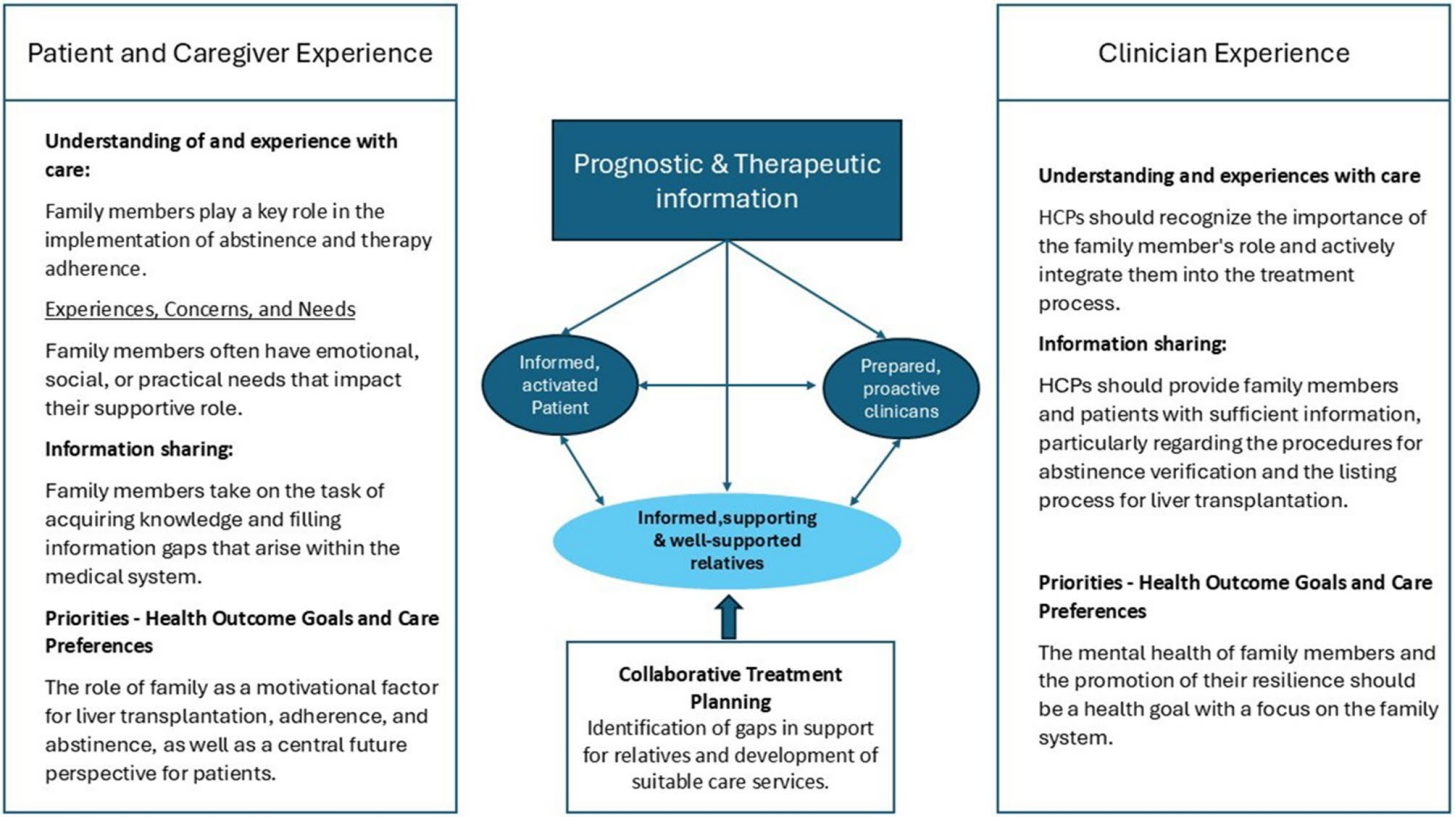
Family-focused IMALD model for LTX (FF-IMALD-LTX)

Finally, we have integrated the findings of our study into the IMALD model by Naik et al. [20] to highlight aspects particularly relevant to family members of patients with alcohol-related cirrhosis prior to liver transplantation in the process of patient-centered care. The integrated aspects with focus on family are shown in Fig. 2, presenting the model adapted to the specific situation “Family-Focused IMALD Model for LTX” (FF-IMALD-LTX).

### Implications for clinical practice

Relatives play a central role in supporting abstinence and treatment implementation prior to liver transplantation. Clinical pathways should therefore systematically integrate family members and address caregiver burden. Family-centered approaches, including CRAFT-informed support [33, 37], may improve both patient stability and caregiver well-being.

### Limitations

This study provides important insights into the experiences and perceived roles of relatives of patients with ALD in the context of liver transplantation. However, several limitations must be acknowledged. Firstly, the recruitment strategy and sample selection may have introduced a self-selection bias, as participants were primarily those who had an interest in the topic or were motivated to share their experiences. As such, perspectives of less-involved or more burdened relatives—and of patients with severe AUD and disrupted family dynamics—may be underrepresented. Secondly, perceptions were assessed through self-report, which can be subject to social desirability bias and limited by the subjective awareness of the participants. Additionally, the insights from HCPs are based on interviews conducted with professionals from 11 liver transplant centers. Although the sample encompassed a range of professional backgrounds, the limited number of participants and the voluntary nature of participation may have resulted in a selection bias, favoring particularly engaged or reflective individuals. As a consequence, the views of HCPs who are less attuned to psychosocial aspects or who work in less collaborative institutional settings may be underrepresented. Lastly, this study was conducted in a single national healthcare context (Germany), which may limit the transferability of findings to countries with different transplant systems or support structures.

Despite these limitations, the study offers valuable initial evidence on the importance of integrating relatives into psychosocial care for patients with ALD and highlights the need for further research in this area, particularly with regard to systemic support structures and cross-national comparisons.

## Supplementary Information

Below is the link to the electronic supplementary material.

Supplementary Material 1

Supplementary Material 2

Supplementary Material 3

## Data Availability

Due to the sensitive nature of the qualitative interview data and the need to protect participant anonymity, the datasets generated and analyzed during the current study are not publicly available. Requests for access to anonymized excerpts for secondary analysis may be considered on a case-by-case basis. Interested researchers should contact the corresponding author. Any data sharing will require prior approval by the local ethics committee and must comply with data protection regulations.

## Abbreviations

ALD: Alcohol-related Liver Disease
CRAFT: Community Reinforcement and Family Training
DGAV: German Society for General and Visceral Surgery
DGVS: German Society for Gastroenterology, Digestive and Metabolic Diseases
HCPs: Health Care Professionals
IMALD: Integrated Model of Advanced Liver Disease
LTX: Liver Transplantation
FF-IMALD-LTX: Family-Focused IMALD Model for LTX
WHO: World Health Organization
