# Supplementary material for: "I am nurse, I am partner, I am cook – I am everything..." roles and functions of relatives in supporting therapy adherence and abstinence in patients with alcohol-related liver cirrhosis prior to listing for liver transplantation: a qualitative analysis
Source: Addict Sci Clin Pract. 2026 May 11;21:44. doi: 10.1186/s13722-026-00673-3 (PMC13169807; doi:10.1186/s13722-026-00673-3)
Supplement: Supplementary file 1 — Supplementary Material 1 [file 13722_2026_673_MOESM1_ESM.docx]

# Supplement Table 1: COREQ Checkliste

| **No.** | **Item** | **Question** | **Answer** |
| --- | --- | --- | --- |
| **Domain 1: Research team and reflexivity** | | | |
| *Personal characteristics* | | | |
| 1 | Interviewer/facilitator | Which author/s conducted the interview or focus group? | ABi: HCPs  JF: Patients & relatives |
| 2 | Credentials | What were the researcher’s credentials? | ABi: MD  JF: Doctoral Student  IL: MD equivalent (without doctoral degree)  ABa: MD, apl. Professor |
| 3 | Occupation | What was their occupation at the time of the study? | ABi: Physician at the Department of Psychotherapy and Psychiatry  JF: Medical student  IL: Physician at the Department of Psychotherapy and Psychiatry  ABa: Head of the Section for Addiction Medicine and Addiction Research |
| 4 | Gender | Was the researcher male or female? | ABi: female  JF: female  IL: male  ABa: male |
| 5 | Experience and training | What experience or training did the researchers have? | ABi: Extensive experience in qualitative research and in conducting qualitative interviews  JF: Formal interviewer training provided by ABi  IL: Longstanding clinical experience in a transplant outpatient clinic  ABa: Extensive expertise in addiction medicine and addiction research |
| *Additional:*  Reflexivity considerations:  The research team consisted of medical researchers at different career stages with clinical and academic backgrounds in psychiatry, psychotherapy, addiction medicine, and transplantation medicine. This expertise supported a nuanced understanding of addiction-related and medical contexts, while also requiring reflection on the potential influence of clinical perspectives on data interpretation.  Interviews were conducted by researchers with formal interview training or extensive research experience. Although no prior personal relationships with participants existed, the recruitment of patients and relatives occurred within a healthcare context, which may have shaped participants’ perceptions and responses. To address this, researchers emphasized their non-clinical role and the independence of the study from patient care.  Reflexivity was maintained throughout the analytic process by critically reviewing emerging categories, discussing interpretations within the research team, and reflecting on alternative readings of the data, including through structured feedback and discussion in an external qualitative research workshop. | | | |
| *Relationship with participants* | | | |
| 6 | Relationship established | Was a relationship established prior to study commencement? | ABi: Two participants (HCPs) were previously acquainted with the interviewer within the wider professional context.  JF: no |
| 7 | Participant knowledge of the interviewer | What did the participants know about the researchers? | ABi: Gender and professional background  JF: Gender and professional background |
| 8 | Interviewer characteristics | What characteristics were reported about the interviewer? | ABi: No additional characteristics reported  JF: No additional characteristics reported |
| **Domain 2: Study design** | | | |
| *Theoretical framework* | | | |
| 9 | Methodological orientation and theory | What methodological orientation was stated to underpin the study? | A qualitative content analysis according to Kuckartz was conducted, as sensitizing concept we use the Integrated Model of Advanced Liver Disease (IMALD) according to Naik et al. (2020)  Stepwise Analytic Procedure:  *Steps 1–6 were conducted for data from patients and relatives by JF, and for HCP data by ABi.*  1. Data preparation and familiarization:  Interview transcripts were prepared and repeatedly read to achieve immersion in the data.  2. Initial text work:  Relevant text segments were identified and annotated with initial notes and memos to capture emerging meanings.  3. Development of main categories:  Main categories were developed in a data-driven manner from the material, reflecting recurrent and salient content.  4 Preliminary coding with main categories:  The entire dataset was coded using the preliminary category system, which was iteratively refined.  5. Development of subcategories:  Subcategories were generated within main categories to capture nuances and variations in meaning.  6. Development of the codebooks:  Two codebooks including category definitions, coding rules, and anchor examples were established.  7. Final coding and consensus process  The finalized codebooks were applied to the datasets by both researchers. In cases of divergent coding, differences were discussed and resolved through consensual coding, resulting in an agreed-upon code.  8. Research-question–guided cross-category analysis:  This step was analytically led by ABi. Categories identified by the research team as relevant to the research question (see Methods section of the main document) were examined in a data-driven, analytically guided manner. Relevant aspects were integrated across categories and thematically structured for the presentation of results. To enhance intersubjective rigor, preliminary findings from this analytic step were presented and discussed in the Qualitative Methods Research Workshop of the Tübingen Center for Public Health and Health Services Research (ZÖGV) at the University Hospital Tübingen with researchers from diverse disciplinary backgrounds and were refined accordingly. |
| 10 | Sampling | How were participants selected? | See Methodes Section |
| 11 | Method of approach | How were participants approached? | Participants (patients with alcohol-related cirrhosis and an indication for liver transplantation, family members, former patients) were recruited through the liver transplantation outpatient clinic, routine telephone contacts, and HCPs via e-mail/telephone. Former patients received a financial incentive of 30€ for participation, while current patients, family members, and HCPs did not receive any compensation. |
| 12 | Sample size | How many participants were in the study? | Patients and Relatives: 24  HCPs: 11 |
| 13 | Non-participation | How many people refused to participate or dropped out? | Patients and relatives were recruited through personal approach as well as via flyers. HCPs were approached via email contacts with individual LTX centres and corresponding email distribution lists. Due to these recruitment methods, the total number of individuals approached could not be determined. In addition, the total number of eligible HCPs across all LTX centres was unknown. Therefore, consent rates could not be calculated.  A total of 20 patient-family pairs were recruited, but not all of them could be included in the actual sample. Of the patients, a total of 4 were willing to participate in an interview and this was also carried out, but communication was so limited due to language barriers, hearing loss and speech impairment following neurological complications that the interviews were not transcribed. Two patients were not interested in participating, while the relatives took part in the interview. For patients whose relatives did not participate in the study, it remained unclear whether the patients did not wish for their relatives to take part, whether the information was unintentionally not passed on, or whether the relatives had no interest in participating. In total, 10 relatives, 3 former patients and 11 patients were included in the study. |
| *Setting* | | | |
| 14 | Setting of the data collection | Where was the data collected? | Patients and relatives: one-on-one settings in the clinic or via phone  HCPs: via telephone |
| 15 | Presence of non-participants | Was anyone else present besides the participants  and researchers? | Patients and relatives: No  HCPs: Brief interruptions at times, e.g., for clinical tasks like phone calls. |
| 16 | Description of sample | Description of sample | The description is provided in the methods section |
| *Data collection* | | | |
| 17 | Interview guide | Were questions, prompts, guides provided by the  authors? Was it pilot tested? | The narrative stimulus and interview guide are available from the authors upon request.  No piloting was conducted; however, the fit was discussed within the team after the first interviews, and no adaptations were required. |
| 18 | Repeat interviews | Were repeat interviews carried out | No |
| 19 | Audio/visual recording | Did the researcher use audio or visual recording to collect the data? | Audio recordings of the interviews were conducted |
| 20 | Field notes | Were field notes made during and/or after the  interview? | Yes, but it was not analyzed separately within the scope of this analysis. |
| 21 | Duration | What was the duration of the interviews? | Patients and relatives: Interviews with those affected lasted between 30-75 minutes (mean 52 minutes), interviews with relatives lasted between 25 and 60 minutes (mean 42 minutes).  HCPs: Expert interviews lasted from 18 to 32 minutes (mean interview time of 26 minutes) |
| 22 | Data saturation | Was data saturation discussed? | Yes, see Methods Section |
| 23 | Transcripts returned | Were transcripts returned to participants for  comment and/or correction? | No. Transcripts were produced by a professional service from audio files and are therefore assumed accurate. |
| **Domain 3: Analysis and findings** | | | |
| *Data analysis* | | | |
| 24 | Number of data coders | How many data coders coded the data? | 2 (ABi & JF); Differences in coding were reconciled by group consensus (whole research team) |
| 25 | Description of the coding tree | Did authors provide a description of the coding  tree? | The coding tree/codebook is not fully presented, as this manuscript reports a cross-analysis based on only selected categories. Instead, the theme definitions from the cross-category analysis are provided in the supplementary material. |
| 26 | Derivation of themes | Were themes identified in advance or derived  from the data? | As an exploratory study, the themes presented in this manuscript were developed data-driven within the cross-category analysis.  (See 9. In this Checklist For details of the analytic process) |
| 27 | Software | What software, if applicable, was used to manage the data? | MAXQDA |
| 28 | Participant checking | Did participants provide feedback on the findings? | No. But for quality assurance purposes, selected excerpts, sections of the codebooks, and parts of the cross-category analysis were presented and discussed in the Qualitative Methods Research Workshop of the Tübingen Center for Public Health and Health Services Research (ZÖGV) at the University Hospital Tübingen. The findings developed in this process were incorporated into the further research process. Consensus meetings among the team members led to the presented results of the in-depth cross- category analysis. |
| *Reporting* | | | |
| 29 | Quotations presented | Were participants’ quotations presented to illustrate the themes/findings? Was each quotation identified? | Yes, illustrative quotes are included in the main text, while additional quotations offering more detailed insights are provided in the supplementary material. |
| 30 | Data and findings consistent | Was there consistency between the data presented and the findings? | Yes |
| 31 | Clarity of major themes | Were major themes clearly presented in the findings? | Definitions of the major themes, which are represented by the headings in the main text, are provided in a table within the supplementary material. |
| 32 | Clarity of minor themes? | Is there a description of diverse cases or  discussion of minor themes? | A detailed overview of the various aspects within the themes is presented in the main text. |
