# Supplementary material for: "I am nurse, I am partner, I am cook – I am everything..." roles and functions of relatives in supporting therapy adherence and abstinence in patients with alcohol-related liver cirrhosis prior to listing for liver transplantation: a qualitative analysis
Source: Addict Sci Clin Pract. 2026 May 11;21:44. doi: 10.1186/s13722-026-00673-3 (PMC13169807; doi:10.1186/s13722-026-00673-3)
Supplement: Supplementary file 2 — Supplementary Material 2 [file 13722_2026_673_MOESM2_ESM.docx]

# Supplement Table 2: Themes, Definitions, and Corresponding Codebook Categories

| Theme | Theme Definition | Codebook Categories |
| --- | --- | --- |
| Implementation and maintenance of therapy adherence | Describes how relatives actively and continuously contribute to the implementation of medical recommendations in everyday life. This includes organizational tasks (arranging and accompanying appointments) as well as practical support with medication management, dietary changes, and maintaining alcohol abstinence. The theme highlights the central role of relatives as a bridge between medical requirements and their application in daily routines, often perceived as essential for treatment success. | - Relatives/Interaction with the patient/Role of relatives  -Lifestyle change/ Alcohol abstinence /Challenges  -Lifestyle change/ Alcohol abstinence /Sobriety  - Lifestyle change/current daily routine/change in diet |
| Acquiring and supporting health literacy | Captures how relatives independently acquire medical and organizational knowledge about liver cirrhosis, LTX, and the healthcare system to better support the affected person. This involves seeking information, communicating with healthcare professionals, and experiencing self-efficacy through knowledge acquisition. The affected individuals themselves tend to adopt a passive role, leaving this responsibility to their relatives. The theme underscores the significance of informed support structures and the shift of knowledge responsibility towards relatives. | - Lifestyle change/current daily routine/change in diet  - Relatives/Dealing with the illness/Emotions |
| Family as a motivating element and future perspective to promote abstinence and adherence | Describes the family—especially children and grandchildren—as a key motivational factor for maintaining alcohol abstinence and treatment adherence. The wish to remain part of family life in the future serves as a driving force for behavioral change. Relatives support this motivation by creating an alcohol-free environment and fostering conditions that promote abstinence. The theme illustrates how family-based future perspectives act as an emotional anchor for health-related behaviors. | - Medical care and support /private support/family  - Relatives/Interaction with the patient/Role of relatives |
| Emotional support and relationship | Encompasses the emotional support provided by relatives and changes in communication patterns and relationship dynamics following diagnosis. This includes daily check-ins, open emotional conversations, and a perceived strengthening of relationships. Adult children, in particular, experience a role reversal, taking on emotional strength and advocacy for their ill parent. The theme shows how emotional closeness and role adaptation can have a stabilizing effect on the illness and transplantation process. | - Relatives/Interaction with the patient/Relationship and communication  - Relatives/Interaction with the patient/Role of relatives  -Relatives/Dealing with the illness/Attitude toward addiction |
| Unmet needs of family members | Highlights the burdens and unmet support needs of relatives. These include time and emotional overload, personal life restrictions, and a lack of organizational and emotional support resources. Relatives report neglecting their own needs and reaching the limits of their energy. The theme makes visible that the support role often comes with significant personal costs that are insufficiently addressed. | - Relatives/interaction with the patient/burdens  - Relatives/Interaction with the patient/Role of relatives |
| Perspective of HCPs on roles and functions of relatives in the process of transplant preparation | Summarizes healthcare professionals’ perspectives on relatives’ roles. HCPs see relatives as essential motivators for abstinence and adherence, recognizing their appreciation of behavioral changes and their importance for long-term post-LTX outcomes. At the same time, they acknowledge relatives’ burdens—particularly those of children—and note the lack of structured support services for them. The theme reflects the dual perception of relatives as indispensable resources and as individuals with their own unmet needs. | - Relatives  - Degree of disclosure/external anamnesis of relatives |
