# Supplementary material for: "I am nurse, I am partner, I am cook – I am everything..." roles and functions of relatives in supporting therapy adherence and abstinence in patients with alcohol-related liver cirrhosis prior to listing for liver transplantation: a qualitative analysis
Source: Addict Sci Clin Pract. 2026 May 11;21:44. doi: 10.1186/s13722-026-00673-3 (PMC13169807; doi:10.1186/s13722-026-00673-3)
Supplement: Supplementary file 3 — Supplementary Material 3 [file 13722_2026_673_MOESM3_ESM.docx]

# **Supplement Table 3 Additional Quotes**

| **Quotes from Patients and Family Members** |
| --- |
| Implementation and maintenance of therapy adherence |
| *Well, I’m basically the one managing appointments.* (11 wife) |
| Acquiring and supporting health literacy |
| *I took everything into my own hands. I pored over books about deceased donation, living donation, requirements — everything*. (15 wife) |
| *I have to take action and really try to get the best out of everything, to find the best possible way — for me, for my husband — to provide support for him wherever I can.* (11 wife) |
| *And it's important that if a doctor comes in now and I say, ‘Could you please call my wife during the ward round? She would like to know what’s going on because I don’t really understand all of this,’ that they actually do it*. (15 patient, in treatment) |
| Family as a motivating element and future perspective to promote abstinence and adherence |
| *I have a daughter. Just for her alone, I want to stay alive. And I also want to stay healthy and get healthy again.* (5 patient, in treatment) |
| *That she suddenly gave up alcohol from one day to the next — that’s true, she really did. Because she’s a family person at heart, and in the end, she only thinks about her family, even now. She was really strict about it — she hasn’t touched a drop since. And we stopped too, because, of course, we had alcohol at home, different kinds of things. But I threw all of it out right away, so that there was really nothing left. And that came from her as well.* (8 husband)  *And that is a kind of support where they accept me for who I am. And they trust me. And that also gives me… well, I don’t want to betray that trust in any way. It’s worth it to me. (8 patient, in treatment)* |
| *The support from my family is very important to me, and I have that support. I don’t think I would have managed as well without it. […] My family is positive. (8 patient, in treatment)* |
| Emotional support and relationship |
| *On the one hand, the fact that my husband always accompanies me means that this has become a shared journey for us. And it’s not just about having someone to talk to — it really helps me a lot.* (1 patient, in treatment) |
| *I think he has started to open up to us more and now tells us when something is on his mind or when he’s not feeling well. In a way, even though the illness was a negative event, it actually brought us closer together and showed us that we are truly there for each other — and that he values having us by his side.* (16 child) |
| *What definitely changed is a bit of a role reversal. Dad has always been the one you go to, the one who represents you, or – it's hard to explain – the one who stands strong in front of you and gives you a shoulder to lean on. And maybe now, he is the fragile one, and we are the ones who have to offer him our shoulder. So, it's kind of a shift. He even says himself that in some areas, we now have to support him more than others would, or more than we would if he were healthy.* (16 child) |
| Unmet Needs of family members |
| *Sometimes, I just wish I could get away more often [vacation trips].* (1 husband) |
| *But for us, the years are coming to an end as well, for me and my wife. We know that. And honestly, our quality of life isn’t great. We’re retired now, have been for four years, my wife and I. And we had other plans, you know?* (5 father)  *We really had to fight for everything ourselves, even for appointments here! […] I really miss having support.* (15 wife) |
| *Yeah, it actually wouldn’t have been a bad idea to talk to the relatives as well.* (3 child) |
| **Quotes from Healthcare Professionals** |
| Perspective of HCPs on roles and functions of relatives in the process of transplant preparation |
| *It is often reported how active alcohol consumption in the past was a significant burden on the family and that relatives now feel relieved. It is seen as a great success that the affected person is now abstinent. Everyone is committed to maintaining this, and relatives play a crucial role in supporting abstinence.* (11HCP psychiatrist) |
| *This can go in both directions – they can be very supportive and helpful, but in some cases, though this is rather the exception, they can also make things more difficult. For example, when relatives drink alcohol themselves. We see this repeatedly – if a partner is not necessarily dependent but regularly consumes alcohol and is unwilling or lacks the insight to reduce or stop drinking for the patient, it can become problematic and create a reinforcing cycle. But overall, relatives are generally very supportive and helpful.* (9HCP psychiatrist) |
| *Relatives are often very positive. The patients we see have already stopped drinking some time ago, and they want the liver transplantation. Naturally, this makes their relatives happy. Most of them are very supportive. […] In chronic cases or when patients have been abstinent for a long time, relatives have often played a significant role, including in the decision to undergo transplantation*. (5HCP psychiatrist) |
| *But when it comes to implementing these measures, I believe that relatives play a significant role. On the one hand, they have a strong interest in ensuring that their loved one receives timely care, and on the other hand, they can also provide essential support. […] Particularly the children of patients awaiting transplantation are often heavily burdened throughout this process and likely require additional support.* (10HCP psychosomatic physician) |
| *I could imagine that relatives also have unmet needs. Because we don’t actually talk to the relatives. We only speak with the patients themselves. Sometimes they are accompanied by family members, but we don’t include them in the conversation. I can very well imagine that they have their own fears and concerns, and that it might be helpful if there were some form of counseling service, a support group, or something similar available for them.* (8HCP psychosomatic physician) |

**Note:** Interviews were numbered consecutively, with patients and their relatives sharing the same number. HCP interviews were numbered separately, using the prefix HCP, followed by by the specialization. Text in square brackets […] indicates that a portion of the interview was omitted here for better readability, without losing any relevant information or it provides additional information from the research team to enhance understanding of a quote.
